# Large left ventricular thrombus in a heavily trabeculated left ventricle

**DOI:** 10.1093/omcr/omae087

**Published:** 2024-08-19

**Authors:** Vasileios Lamprou, Simon G Williams, Raana Alamgir, Paul Callan

**Affiliations:** North West Heart Centre, Wythenshawe Hospital, Manchester University NHS Foundation Trust, Southmoor Road, Manchester, M23 9LT, United Kingdom; North West Heart Centre, Wythenshawe Hospital, Manchester University NHS Foundation Trust, Southmoor Road, Manchester, M23 9LT, United Kingdom; The University of Manchester, Oxford Road, Manchester, M13 9PL, United Kingdom; North West Heart Centre, Wythenshawe Hospital, Manchester University NHS Foundation Trust, Southmoor Road, Manchester, M23 9LT, United Kingdom; North West Heart Centre, Wythenshawe Hospital, Manchester University NHS Foundation Trust, Southmoor Road, Manchester, M23 9LT, United Kingdom

## Case report

We present a case of a 49-year-old Caucasian male with an established diagnosis of left ventricular non compaction cardiomyopathy (LVNC) since 2006. An initial referral for transplant assessment was due to severe left ventricular (LV) dilation and dysfunction. However, as he was asymptomatic on optimal medical therapy he was discharged to the care of his local heart failure services.

He remained clinically well until December 2023 when he had four hospital admissions within three months and was referred back to our transplant centre. Prior to transfer he developed an episode of pulseless ventricular tachycardia, from which he was resuscitated and commenced on inotropes.

Transthoracic echocardiogram (TTE) cines demonstrate 2 mobile structures 1.8 cm × 1.3 cm and 1.1 × 0.9 cm held between prominent trabeculation (Video 1). Appearances are consistent with thrombi (Video 2).

He was started on warfarin with bridging of tinzaparin until INR was above 2.

He remained unwell with multi-organ failure despite inotropic support, and he received a biventricular assist device (BiVAD) as a bridge to transplant. Due to extensive LV thrombus, the inflow cannula was placed in the left atrium, which led to flow issues, and generated further clot. The patient developed an extensive embolic stroke in the middle cerebral artery, which is an absolute contraindication for transplant and after multidisciplinary assessment and discussion with the family, treatment was withdrawn.

## Discussion

Left ventricular noncompaction is a rare cardiomyopathy characterized by a thin, compacted epicardial layer and a noncompacted endocardial layer [1].

Major clinical sequalae of LVNC are arrhythmias, heart failure and thromboembolic events. Prominent trabeculae and deep recesses form substrate for slow flowing blood and risk for thrombus formation [1, 2].

Although there is controversy about prophylactic anticoagulation, studies have supported to systematically anticoagulate patients with atrial fibrillation, previous thromboembolic events, and ejection fraction less than 40% [3]. Direct oral anticoagulants (DOAC) can be used as 1^st^ line prophylactic treatment and Vitamin K antagonists (VKA) should be considered as 1^st^ choice for patients with LV thrombus [1, 3].

## Supplementary Material

Video_1_omae087

Video_2_omae087
